# Ethnocultural differences in symptom change, engagement, and adherence in internet-delivered cognitive behavioral therapy: A secondary analysis of a healthcare-embedded longitudinal study

**DOI:** 10.1016/j.invent.2026.100974

**Published:** 2026-07-09

**Authors:** Noa Roemmel, Heather D. Hadjistavropoulos, Marcie Nugent, Roselind Lieb, Gunther Meinlschmidt

**Affiliations:** a Department of Digital and Blended Psychosomatics and Psychotherapy, Psychosomatic Medicine, University Hospital Basel, Basel, Switzerland; b Faculty of Medicine, University of Basel, Basel, Switzerland; c Division of Clinical Psychology and Epidemiology, Faculty of Psychology, University of Basel, Basel, Switzerland; d Online Therapy Unit, Department of Psychology, University of Regina, Regina, Saskatchewan, Canada; e Department of Psychology, Clinical Psychology and Psychotherapy – Methods and Approaches, University of Trier, Trier, Germany

**Keywords:** Internet-based intervention, Cognitive behavioral therapy, Psychotherapy, Ethnocultural groups, Engagement, Treatment outcome

## Abstract

**Background:**

Internet-delivered cognitive behavioral therapy (iCBT) is a scalable approach to address the mental health treatment gap. Its effectiveness for People of Diverse Ethnocultural Backgrounds (PDEGs) remains uncertain due to persistent underrepresentation in research and routine care.

**Objective:**

This secondary analysis examined ethnocultural differences in symptom change, engagement, and adherence in a healthcare-embedded iCBT program, and whether engagement or adherence mediated ethnocultural differences in treatment outcomes.

**Methods:**

Data from 2604 adults receiving guided iCBT for depression and anxiety through the Online Therapy Unit in Saskatchewan, Canada (February 2020–December 2021) were analyzed. Participants were categorized as White (n = 2281) or PDEGs (n = 323; multiple ethnocultural subgroups). Engagement metrics included logins, therapist messages, and phone contacts. Adherence metrics included lessons completed, program completion (≥4 lessons), and weekly questionnaire submission. Depression and anxiety were assessed using the Patient Health Questionnaire-9 and Generalized Anxiety Disorder-7. Regression and mixed-effects models compared outcomes across groups, controlling for age, dataset, and baseline symptoms. Exploratory mediation analyses were conducted.

**Results:**

Both groups showed significant reductions in depression and anxiety, with no differences in symptom improvement. Engagement was comparable. However, PDEGs were less likely to complete the program. Mediation analyses did not indicate engagement or adherence explained outcome differences.

**Conclusions:**

Our findings did not indicate differences in symptom improvement or engagement between White participants and PDEGs, although aggregation due to small subgroup sizes may have obscured subgroup-specific differences. Modest adherence differences and PDEG underrepresentation highlight the need for further research to understand barriers to program completion and equitable access to iCBT.

## Highlights

• iCBT was associated with reductions in depression and anxiety across ethnocultural groups.
• PDEGs appeared to have slightly lower program completion rates than White participants.
• Engagement and adherence did not mediate ethnocultural differences in outcomes.
• PDEG underrepresentation underscores the need to further examine barriers to equitable iCBT access.

## 1 Background

The global burden of anxiety and depressive disorders is rising, underscoring the urgent need for scalable mental health interventions (Zhang et al., 2026). Given this growing demand for mental health care, digital mental health interventions (DMHI) have emerged as effective, low-threshold treatments (Balcombe and De Leo, 2021; Hedman-Lagerlöf et al., 2023). Among these, internet delivered cognitive behavioral therapy (iCBT), namely online structured CBT modules often supported by therapist guidance via secure messaging or telephone, is one of the most extensively studied approaches (Andersson et al., 2019). Compared to face-to-face psychotherapy, iCBT offers advantages in scalability, accessibility, and reduced stigma (Balcombe and De Leo, 2021; Andersson and Titov, 2014), with potential to improve reach among individuals less likely to access conventional services, including younger people, socioeconomically disadvantaged groups, and ethnic minorities (Andersson and Titov, 2014; Willis et al., 2022).

Prior research indicates that although unmet mental health care needs are substantial across populations, they are disproportionately higher among ethnic minorities (Chiu et al., 2018), particularly younger individuals (Lu et al., 2021; Ma et al., 2025). Evidence from Canada shows that barriers to mental health care vary across ethnic groups and conditions, with Indigenous peoples consistently reporting the highest treatment barriers, followed by Black Canadians, while Asian groups report the lowest (Williams et al., 2024). These findings mirror broader ethnicity-based disparities in prevalence and help-seeking. Differences across ethnic groups and specific disorders are shaped by unequal access and health service resource gaps. In this study, we use the term People of Diverse Ethnocultural Backgrounds (PDEGs) to encompass Indigenous Canadians and racialized minorities, including Black, Asian, and Latin American populations, aligning with the broader construct often referred to as Black, Indigenous, and People of Color (BIPOC) in the literature (Sapkota et al., 2023; Sapkota et al., 2024).

Despite evidence of PDEGs and White individuals showing similar willingness to engage with DMHI, structural barriers to equitable access persist (Lorenzo-Luaces et al., 2024; Stiles-Shields et al., 2022). For example, a six-year analysis of iCBT use in Saskatchewan (2013–2019) showed consistently lower participation among PDEGs (∼10%) compared to their representation in the province's total population (∼32%) (Sapkota et al., 2024; Hadjistavropoulos et al., 2022a). In fact, the majority of iCBT users resemble those in traditional face-to-face therapy, who are more likely to be female, White, and of higher socioeconomic status (Knipe et al., 2025; Ramos and Chavira, 2022; Aemissegger et al., 2022).

Research on ethnic disparities in DMHI uptake and engagement is scarce, as PDEGs are often underrepresented or unreported in iCBT trials, resulting in limited evidence on their engagement, adherence, and treatment outcomes (Andrews et al., 2018; De Jesús-Romero et al., 2024; Kalmbach et al., 2023). Hence, findings on ethnocultural differences in iCBT engagement patterns remain mixed. For example, one study reported that PDEGs accessed significantly fewer modules than White participants (Solar et al., 2021). Evidence from a meta-analysis also indicates that ethnicity moderates treatment outcomes, with minoritized groups showing lower response and remission rates than majority populations, underscoring the need for tailored interventions to improve effectiveness (Karyotaki et al., 2018). However, other studies report no significant ethnocultural differences in iCBT engagement and treatment outcome (Cheng et al., 2019; Owusu et al., 2023).

Furthermore, evidence on the effectiveness of cultural adaptations in mental health interventions remains inconclusive (Benish et al., 2011; Huey and Polo, 2008; Hall et al., 2016), limited and inconsistent findings in DMHIs (Ramos and Chavira, 2022; Ellis et al., 2022). Notably, one observational trial reported comparable engagement, satisfaction, and treatment outcomes between culturally adapted and non-adapted iCBT among PDEGs (Sapkota et al., 2024).

Taken together, these findings underscore both the promise and challenges of iCBT for PDEGs. Although iCBT is efficacious in general populations, evidence for PDEGs remains mixed, with underrepresentation in trials and indications of higher dropout, lower engagement, and reduced symptom improvement (Chiu et al., 2018; Aemissegger et al., 2022; De Jesús-Romero et al., 2024; Karyotaki et al., 2018). Engagement and program adherence are believed to be associated with treatment benefit (Gan et al., 2021). Notably, a recent systematic review and meta-analysis demonstrated that greater engagement, particularly higher module completion, was modestly but significantly linked to improved outcomes across conditions and guidance levels (Gan et al., 2021). Thus, ethnocultural disparities in outcomes may reflect differential engagement rather than solely the absence of cultural adaptation. Given inconsistent findings on cultural adaptations (Ellis et al., 2022), clarifying the role of engagement in shaping ethnocultural differences in treatment outcomes remains a key research priority.

### 1.1 Objective

This study investigates the role of engagement and adherence in shaping ethnocultural differences in iCBT. Specifically, we investigate (1) whether PDEGs differ from White participants in symptom change, engagement, and adherence, and (2) whether observed engagement or adherence differences mediate ethnocultural disparities in treatment outcomes.

## 2 Methods

### 2.1 Study design and setting

**Fig. 1:**
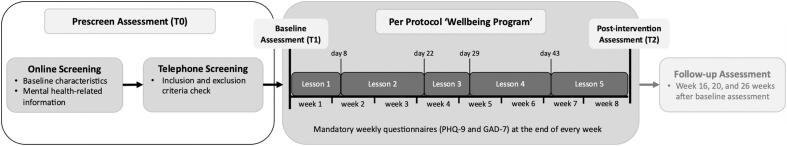
Overview of the Online Therapy Unit (OTU) Wellbeing Course structure. Participants first completed online and telephone screening before accessing the 8-week internet-delivered cognitive behavioral therapy (iCBT) program. The program included five sequential lessons, weekly symptom questionnaires, and post-intervention assessment at week 8. Follow-up assessments were not included in the present analysis. GAD-7 = Generalized Anxiety Disorder-7; iCBT = internet-delivered cognitive behavioral therapy; OTU=Online Therapy Unit; PHQ-9 = Patient Health Questionnaire-9.

In this secondary analysis we used a subset of participants described in our previous work (Roemmel et al., 2025). Data was drawn from multiple longitudinal studies conducted within the empirically supported transdiagnostic iCBT program called the “Wellbeing Course” delivered by the Online Therapy Unit (OTU) at the University of Regina, Canada (Hadjistavropoulos and Peynenburg, 2025) (including two registered trials: NCT04228575 and NCT04512768). The program is integrated into routine healthcare and follows the Learning Health System (LHS) framework (Menear et al., 2019), ensuring the integration of data-based improvement of safety, quality, and treatment effectiveness with routine patient care. For detailed descriptions of the Wellbeing Course, we refer to Fig. 1, Online Supplement 1, and earlier publications from this project (Titov et al., 2015a; Titov et al., 2015b).

### 2.2 Ethical considerations

The iCBT program and its associated studies adhere to the ethical principles outlined in the Declaration of Helsinki, as well as the Human Research Act (HRA) and Human Research Ordinance (HRO). This secondary analysis received ethical approval from the Research Ethics Board at the University of Regina (REB 2022-140, approved on 2023-01-10) and the Ethics Committee of Northwest and Central Switzerland (EKNZ 2022-02263, approved on 2023-01-03). We obtained informed consent from all participants, and all data were de-identified prior to storage and analysis at the sciCORE scientific computing center of the University of Basel (sciCORE, 2021). This study was conducted under a data-sharing agreement between the University of Regina and the University Hospital Basel.

### 2.3 Inclusion criteria and recruitment

Eligibility for the OTU Wellbeing Course requires adults (≥18 years) living in Saskatchewan, Canada who are experiencing at least mild symptoms of depression or anxiety, operationalized as scoring 5 or higher on the Patient Health Questionnaire-9 (PHQ-9, (Kroenke et al., 2001)) or Generalized Anxiety Disorder-7 (GAD-7, (Spitzer et al., 2006)) at pre-screen assessment. Participants must also have reliable internet access, basic digital literacy, and provide contact information for a healthcare professional who can be reached in urgent situations. A psychiatric diagnosis is not required. Individuals are excluded if they present with acute suicide risk, have recently attempted suicide or been psychiatrically hospitalized, display unmanaged psychosis or mania, report primary problem with severe substance use, or attend more than two in-person therapy sessions per month. Those unwilling to engage with iCBT are also ineligible. Patients learn about the OTU through clinician referrals, media, family or friends, and online advertisements (Hadjistavropoulos et al., 2022a). For this study, we included all participants who enrolled in the ‘Wellbeing Course’ between February 2020 and December 2021, provided consent for the use of their data for program evaluation, initiated treatment by accessing at least the first lesson, and completed baseline assessment. This time frame was selected because the administration of weekly questionnaires was consistent throughout this period, while assessment protocols varied prior to February 2020.

### 2.4 Study variables

#### 2.4.1 Symptom change and treatment outcome

In this study, depressive and anxiety symptoms were assessed using total scores on the PHQ-9 and GAD-7 at prescreen (week 0) and weekly throughout the program, with week 8 as post-intervention. The primary outcome was symptom change from week 0 to week 8, examined using longitudinal symptom data across weeks 0–8. Supportive analyses additionally examined symptom change from week 0 to week 8 using continuous change scores and binary indicators of clinically significant improvement. Clinically significant improvement was defined as a reduction of ≥5 points on the PHQ-9 (Kroenke, 2012) and ≥4 points on the GAD-7 (Toussaint et al., 2020). Participants who did not meet these thresholds were classified into the “other” category, reflecting no clinically significant change. Clinically significant deterioration was not examined separately due to the low number of cases, particularly among PDEGs.

#### 2.4.2 Engagement

Engagement was operationalized using objective platform-based metrics, including the total number of logins, as well as guidance metrics, namely total phone contacts with clinicians, and total secure messages sent and received (all integer counts). Further details regarding therapist support procedures, including secure messaging and telephone contact, are provided in Online Supplement 1. Engagement metrics reflect patient-level totals across the period of platform use. Therefore, these metrics do not capture when engagement occurred or how it was distributed over the course of the program, and patients may have engaged for fewer or more than 8 weeks (e.g., completing all lessons in a shorter period or engaging longer without completing all lessons).

#### 2.4.3 Program adherence

Adherence was captured as (a) weekly PHQ-9 and GAD-7 questionnaire submission (binary, completed vs. not completed), (b) total number of weeks with PHQ-9 and GAD-7 questionnaire completion (integer count), (c) lesson completion, recorded as the total number of lessons accessed (1–5), and (d) a binary indicator of program completion, defined as accessing at least four lessons (≥4 lessons), since the 5th lesson was considered repetition and relapse prevention. Of note, patients were not able to continue working on lesson content if they did not complete the corresponding weekly questionnaire.

#### 2.4.4 Ethnicity

Ethnicity was assessed as a binary variable, distinguishing participants who self-identified as White (Caucasian) from those self-identified as PDEGs, including minoritized groups (First Nations, Inuit, Métis; Asian; South Asian; Black; Latin American; Middle Eastern; or Other backgrounds). This terminology follows conventions used in previous OTU research (Sapkota et al., 2024) and aligns with populations commonly referred to as BIPOC, a term often used to describe visible and racialized minority groups.

#### 2.4.5 Covariates

Based on prior findings indicating their relevance for iCBT engagement and outcomes (Edmonds et al., 2018), age (in years) and prescreen (T0) symptom severity (PHQ-9 and GAD-7 total scores) were included as covariates. We used prescreen (T0) PHQ-9 and GAD-7 scores instead of baseline scores collected at program entry to ensure temporal precedence and avoid conditioning on variables potentially influenced by early program engagement. Since this secondary analysis used pooled longitudinal data drawn from multiple studies within the iCBT Wellbeing Course delivered by the OTU program, we included dataset as a covariate to account for potential heterogeneity in study focus, recruitment procedures, and implementation context across datasets, ensuring that estimated symptom trajectories and group effects reflect within-participant change rather than between-study variation. Although the content of the 8-week Wellbeing course remained consistent, some participants were enrolled in parallel or nested studies that included adjunct intervention components. These included a sleep-focused randomized controlled trial that provided supplementary sleep-related content at pre-treatment (ClinicalTrials.gov NCT04512768, Sleep Randomized Controlled Trial (RCT) (Edmonds et al., 2024)), a factorial trial that offered an optional booster lesson and extended therapist support for up to 12 weeks after post-treatment (ClinicalTrials.gov NCT04228575, Booster/Extension Randomized Factorial Trial (RFT) (Hadjistavropoulos et al., 2022b)), and an observational study where patients were provided access to additional alcohol-related resource materials (Peynenburg et al., 2023). These components were delivered as adjuncts and were not the focus of this analyses. Further details are provided in the associated publications.

### 2.5 Statistical analyses

#### 2.5.1 Data preparation

All computations were performed on sciCORE at the University of Basel (sciCORE, 2021). Statistical analyses and visualizations were carried out in R using RStudio 2024.03.999-dev + 999 “Ocean Storm” (Linux), operated in a Mozilla/5.0 environment (see Online Supplement 2 for details). Analyses were restricted to participants enrolled after February 2020 to ensure consistency in program content and data collection procedures. Prior to modelling, variables were inspected for missingness, range and distributional irregularities, and reshaped as needed for longitudinal analyses of weekly symptom data.

#### 2.5.2 Sociodemographic characteristics and mental health history

We used descriptive statistics to characterize the sample and compare White and PDEG participants on sociodemographic characteristics, prescreen symptom severity, and mental health history. We tested group differences using *t*-tests for continuous variables and *χ*^*2*^ tests for categorical variables. Effect sizes (Hedges' *g* for continuous variables, Cramér's *V* for categorical variables) were reported to quantify the magnitude of between-group differences. In addition, we computed a Pearson correlation matrix to assess associations among engagement and adherence metrics and visualized them as a heatmap.

#### 2.5.3 Analyses addressing research question (RQ) 1: group differences in symptom change, engagement, and adherence

To examine whether symptom change from prescreen (T0) to post-intervention at week 8 (T2) differed between PDEGs and White participants, we fitted linear mixed-effects models separately for PHQ-9 and GAD-7 scores across weeks 0–8. Models included fixed effects for week (coded 0–8), ethnicity (PDEGs vs White participants), and their interaction, with random intercepts and random slopes for week at the participant level. For each outcome, we adjusted models for prescreen symptom severity (prescreen PHQ-9 for PHQ-9 outcomes and prescreen GAD-7 for GAD-7 outcomes). Cross-symptom prescreen covariates (prescreen GAD-7 in PHQ-9 models and prescreen PHQ-9 in GAD-7 models) were not included to avoid overadjustment and multicollinearity and to ensure that model estimates reflect change over time in the focal symptom domain rather than prescreen comorbidity.

The primary parameter of interest was the week × ethnicity interaction, which tests whether the rate of symptom change over time differed by ethnocultural group. Models were estimated using maximum likelihood and included age, dataset, and prescreen symptom severity as covariates. Linear mixed-effects models were used as the primary approach because they estimate group differences in symptom trajectories across repeated weekly assessments while incorporating all available observations under a missing-at-random assumption, thereby limiting bias relative to complete-case analyses when missingness depends on observed data. To assess whether post-intervention (T2) missingness differed by ethnocultural group, we compared rates of missing PHQ-9 and GAD-7 scores at week 8 between White participants and PDEGs using *χ*^*2*^ tests. In addition, we examined whether post-intervention (T2) symptom missingness was associated with prescreen symptom severity and observed engagement and adherence indicators using logistic regression models to assess the plausibility of a missing-at-random assumption.

Clinically significant improvement from week 0 to week 8 was analyzed using logistic regression models with ethnicity as the primary predictor. Adjusted models included age, dataset, and prescreen symptom severity, and results are reported as odds ratios with 95% confidence intervals (CI). Within-group changes in PHQ-9 and GAD-7 from week 0 to week 8 were examined descriptively using paired-samples *t*-tests restricted to participants with complete data at both time points. Observed weekly symptom trajectories were summarized using group means and 95% CI based on available data.

Distributions of engagement and adherence variables were examined using histograms and model residual diagnostics prior to analysis. We analyzed continuous engagement indicators (e.g., number of logins, guidance contacts, messages exchanged) using linear regression models adjusted for age, dataset, and prescreen PHQ-9 and GAD-7 scores, with heteroskedasticity-consistent (HC3) Huber-White robust standard errors. Adjusted means were estimated at the sample mean of the covariates.

For the evaluation of program adherence, we used three indicators: program completion (≥4 lessons completed; binary), the total number of lessons completed (0–5; continuous), and the number of weeks with any PHQ-9 or GAD-7 submissions (continuous). Program completion was analyzed using both a logistic regression model to obtain adjusted odds ratios and a linear probability model to estimate adjusted probability differences, and a modified Poisson model with log link and HC3 robust standard errors to estimate adjusted risk ratios. Total lessons completed were analyzed using ordinary least squares regression. All models included ethnicity (PDEGs vs. White participants), and age, dataset, and prescreen PHQ-9 and GAD-7 as covariates. We used HC3 robust standard errors for the linear models and for the logistic regression.

Moreover, we examined weekly questionnaire submission (PHQ-9 or GAD-7 submitted vs. not submitted) during program weeks 1–8 using a generalized linear mixed-effects model (GLMM) with a binomial link. Week and continuous covariates were standardized prior to model estimation. The model included random intercepts and random slopes for week at the participant level, and fixed effects for week, ethnicity, and their interaction, adjusting for age, dataset, and prescreen (T0) PHQ-9 and GAD-7 scores. This framework evaluated ethnic differences in adherence trajectories across the treatment period while accounting for within-participant correlation and prescreen clinical severity.

#### 2.5.4 Analyses addressing RQ2: mediation of ethnocultural differences in treatment outcomes through engagement and adherence

To address Research Question 2 (RQ2), whether engagement and program adherence mediate ethnocultural differences in treatment outcome, we used an Analysis of Covariance (ANCOVA)-based mediation framework with post-intervention (T2) PHQ-9 and GAD-7 scores as separate outcomes. These analyses were exploratory given the small overall ethnocultural differences in post-intervention symptoms, but were conducted because previous research has linked engagement with digital interventions to mental health outcomes (Gan et al., 2021). Ethnocultural group (PDEGs vs. White participants) was entered as the predictor, and five engagement indicators (days with platform access, number of logins, messages sent, messages received, and phone conversations) and three adherence indicators (number of weeks with any PHQ-9 or GAD-7 submitted, program completion defined as ≥4 lessons, and total lessons completed) were each evaluated as single mediators in separate models.

We used the mediation package in R to implement the ANCOVA. For adjusted models, each mediator was regressed on ethnicity, age, dataset, and prescreen (T0) PHQ-9 or GAD-7 severity, similarly the post-intervention (T2) outcomes were regressed on ethnicity, the mediator, age, dataset and prescreen (T0) symptom severity. From these models we estimated the indirect effect (ACME) with 95% CI obtained via simulation. Given the observational nature of engagement and adherence as well as the absence of randomization to mediator levels, effects are interpreted as model-based decompositions of associations under the ANCOVA specification rather than causal effects.

Engagement and adherence indicators included count-type variables (e.g., logins, messages, weeks submitted, lessons completed) and a binary variable (program completion ≥4 lessons). We examined distributions for skewness and zero-inflation and, for interpretability and consistency across mediators, estimated mediation models using linear regressions as primary analyses (ANCOVA specification). Because linear models may be sensitive to non-normality for count/binary mediators, we conducted sensitivity checks using alternative specifications (e.g., log(1 + x) transformations for highly skewed counts and/or generalized linear models for binary/count mediators where appropriate) and verified that substantive conclusions did not change.

Because post-intervention (T2) outcomes and several covariates were partially missing, we conducted both complete-case and multiple-imputation (MI) analyses. We restricted complete-case analyses to participants with observed post-intervention (T2) PHQ-9 and GAD-7. For MI, we used the *mice* package with 20 imputations and predictive mean matching for continuous variables to impute missing age, prescreen PHQ-9 and GAD-7, and post-intervention (T2) PHQ-9 and GAD-7 scores. The imputation model included ethnicity, all weekly PHQ-9 and GAD-7 scores (weeks 1–8), last-observation-carried-forward PHQ-9 and GAD-7 (included only as auxiliary predictors), engagement and adherence variables, and sociodemographic and mental-health-history variables. Participant ID and ethnicity were not imputed. Mediator variables were not imputed as engagement and adherence indicators were derived from platform-use and program records. Therefore, missing values were interpreted as absence of observed engagement or adherence and coded as zero. This operationalization was applied consistently across complete-case and multiple-imputation analyses. As sensitivity analysis we compared complete-case, unadjusted MI, and MI models adjusted for age, dataset, and prescreen (T0) PHQ-9 and GAD-7 scores.

#### 2.5.5 Additional analyses

In exploratory analyses, we examined whether symptom outcomes, engagement, and adherence differed across ethnocultural subgroups relative to White participants. Analyses were restricted to subgroups with adequate sample size (≥20 participants), with White participants serving as the reference group. For each subgroup contrast, we estimated separate linear regression models (see Online Supplement 3 for details).

## 3 Findings

### 3.1 Participant characteristics

This secondary analysis included *N* = 2643 patients who participated in the OTU program between February 2020 and December 2021. A total of *n* = 39 (1.5%) patients did not report their ethnicity, resulting in ethnicity data for *n* = 2604 patients. Of these, *n* = 2281 (88%) identified as White and 323 (12%) as PDEGs. Patients identifying as PDEGs comprised of *n* = 167 (51.7%) First Nations, Inuit, Metis, Asian *n* = 45 (13.9%), Other *n* = 36 (11.2%), South Asian *n* = 27(8.4%), Black *n* = 18 (5.6%), Latin American *n* = 17 (5.3%), and Middle Eastern *n* = 13 (4.0%). Sample characteristics and prescreen (T0) depressive and anxiety symptoms are presented in Table 1. Comparisons between White and PDEG participants indicated significant differences with small effect sizes in age, employment status, location and prescreen (T0) PHQ-9 scores, with White participants being older, more likely to have paid work and less likely to still be in school.

**Table 1:** Sample characteristics.

	Statistic	Full sample *N* = 2604		White participants *n* = 2281		PDEGs *n* = 323		*p*-value^1^	Effect size
Age in years	Mean (SD)	37	(13.0)	38	(14.0)	34	(11.0)	<0.001	0.27 (*g*)
Missing	n (%)	6	(0.2)	5	(0.2)	1	(0.3)		
Gender	n (%)							0.259	0.03 (*V*)
Women		1995	(77.0)	1751	(77.0)	244	(76.0)		
Men		577	(22.0)	505	(22.0)	72	(22.0)		
Non-binary, transgender, two-spirit, not listed, or prefer not to disclose		32	(1.2)	25	(1.1)	7	(2.2)		
Missing		0	(0)	0	(0)	0	(0)		
Highest education	n (%)							0.147	0.06 (*V*)
High school diploma		460	(18.0)	396	(17.0)	64	(20.0)		
Some college or university		726	(28.0)	642	(28.0)	84	(26.0)		
College diploma (2 to 3 year)		459	(18.0)	405	(18.0)	54	(17.0)		
University undergraduate degree		643	(25.0)	572	(25.0)	71	(22.0)		
University graduate degree (e.g. MA, PhD)		148	(5.7)	122	(5.3)	26	(8.0)		
University professional degree (e.g. MD)		95	(3.6)	78	(3.4)	17	(5.3)		
Other		73	(2.8)	66	(2.9)	7	(2.2)		
Missing		0	(0)	0	(0)	0	(0)		
Relationship status	n (%)							0.012	0.08 (*V*)
Dating		304	(12.0)	261	(11.0)	43	(13.0)		
Living with partner		195	(7.5)	167	(7.3)	28	(8.7)		
Married or common law		1375	(53.0)	1228	(54.0)	147	(46.0)		
Separated		94	(3.6)	82	(3.6)	12	(3.7)		
Divorced/Widowed		143	(5.5)	131	(5.7)	12	(3.7)		
Single never married		493	(19.0)	412	(18.0)	81	(25.0)		
Missing		0	(0)	0	(0)	0	(0)		
Having at least one child	n (%)	1440	(55.0)	1279	(56.0)	161	(50.0)	0.041	0.04 (*V*)
Missing		0	(0)	0	(0)	0	(0)		
Employment status	n (%)							<0.001	0.10 (*V*)
I am still at school		148	(6.6)	112	(5.8)	36	(13)		
I have paid work		1378	(62.0)	1227	(63.0)	151	(53)		
I run the household (and, if applicable, care for children)		440	(20.0)	386	(20.0)	54	(19)		
I am (partially) unfit for paid work because of health problems		264	(12.0)	222	(11.0)	42	(15)		
Missing		374	(14.4)	334	(14.6)	40	(12.4)		
Disability benefits (last 3 months)	n (%)	157	(9.4)	134	(9.1)	23	(12)	0.270	0.03 (*V*)
Missing		929	(35.7)	801	(35.1)	128	(39.6)		
Residential Location	n (%)							0.003	0.09 (*V*)
Small town (200–800 citizens)		123	(4.7)	108	(4.7)	15	(4.6)		
Town (800–7000 citizens)		280	(11.0)	255	(11.0)	25	(7.7)		
Big town (7000–20,000 citizens)		135	(5.2)	120	(5.3)	15	(4.6)		
Small city (20,000–100,000 citizens)		249	(9.6)	205	(9.0)	44	(14.0)		
City (100,000–300,000 citizens)		1269	(49.0)	1098	(48.0)	171	(53.0)		
Large city (300,000+ citizens)		210	(8.1)	181	(7.9)	29	(9.0)		
Other		338	(13.0)	314	(14.0)	24	(7.4)		
Missing		0	(0)	0	(0)	0	(0)		
How participants heard about the program	n (%)							0.27	0.05 (*V*)
Employer, Union, Work, Colleague, Professional Association		84	(3.2)	74	(3.2)	10	(3.1)		
Friend/family member		379	(15.0)	335	(15.0)	44	(14.0)		
Media (e.g., newspaper, radio, TV)		25	(1.0)	25	(1.1)	0	(0)		
Mental health professional or health region intake		296	(11.0)	265	(12.0)	31	(9.6)		
Online source (e.g. website or email)		225	(8.6)	189	(8.3)	36	(11.0)		
Physician or other medical health professional		1498	(58.0)	1308	(57.0)	190	(59.0)		
Printed poster/other		97	(3.7)	85	(3.7)	12	(3.7)		
Missing		0	(0)	0	(0)	0	(0)		
Dataset	n (%)							0.885	0.01 (*V*)
Booster/extension RFT (NCT04228575)		851	(33)	742	(33)	109	(34)		
Additional alcohol-related materials (OS)		1357	(52)	1190	(52)	167	(52)		
Sleep RCT (NCT04512768)		396	(15)	349	(15)	47	(15)		
Missing		0	(0)	0	(0)	0	(0)		
PHQ-9 at prescreen (T0)	Mean (SD)	13.6	(5.6)	13.5	(5.6)	14.3	(5.8)	0.026	−0.14 (*g*)
PHQ-9 severity at prescreen (T0)	n (%)							0.214	0.05 (*V*)
None/minimal (0–4)		130	(5.0)	116	(5.1)	14	(4.3)		
Mild (5–9)		563	(22.0)	500	(22.0)	63	(20.0)		
Moderate (10–14)		755	(29.0)	670	(29.0)	85	(26.0)		
Moderately severe (15–19)		706	(27.0)	614	(27.0)	92	(28.0)		
Severe (20–27)		450	(17.0)	381	(17.0)	69	(21.0)		
Missing		0	(0)	0	(0)	0	(0)		
GAD-7 at prescreen (T0)	Mean (SD)	12.7	(5.0)	12.6	(5.0)	13.2	(4.9)	0.075	−0.10 (*g*)
GAD-7 severity at prescreen (T0)	n (%)							0.469	0.03 (*V*)
None/minimal (0–4)		128	(4.9)	117	(5.1)	11	(3.4)		
Mild (5–9)		618	(24.0)	546	(24.0)	72	(22.0)		
Moderate (10–14)		833	(32.0)	727	(32.0)	106	(33.0)		
Severe (15–21)		1025	(39.0)	891	(39.0)	134	(41.0)		
Missing		0	(0)	0	(0)	0	(0)		
Previous mental health treatment									
Currently receiving minimal MH treatment	n (%)	1125	(43.0)	1000	(44.0)	125	(39.0)	0.092	0.03 (*V*)
Missing		0	(0)	0	(0)	0	(0)		
Currently on MH waitlist	n (%)	292	(11.0)	257	(11.0)	35	(11.0)	0.892	0.0 (*V*)
Missing		0	(0)	0	(0)	0	(0)		
Currently taking psychotropic medication		1455	(56.0)	1293	(57.0)	162	(50.0)	0.031	0.04 (*V*)
Missing		0	(0)	0	(0)	0	(0)		
Ever took psychotropic medication (lifetime)	n (%)	1863	(72.0)	1650	(72.0)	213	(66.0)	0.020	0.05 (*V*)
Missing		0	(0)	0	(0)	0	(0)		
Any current or past medical problems	n (%)	1273	(49.0)	1118	(49.0)	155	(48.0)	0.775	0.01 (*V*)
Missing		0	(0)	0	(0)	0	(0)		

**Notes.** Percentages are calculated within each ethnocultural group (column percentages), allowing comparison between White participants and PDEGs. Missing values are shown separately. Sample restricted to participants with non-missing ethnicity (*N* = 2604). Dataset (OTU study version) indicates the specific study within the Online Therapy Unit program from which each participant's data originated. Effect size: Hedges' g for continuous variables; Cramer's V for categorical variables. Benchmarks: g ≈ 0.2 small, 0.5 medium, 0.8 large; V ≈ 0.1 small, 0.3 medium, 0.5 large. *P*-values compare White participants vs PDEGs. Abbreviations: GAD-7 = Generalized Anxiety Disorder-7; MH = Mental Health; OS=Observational Study; PDEGs = People of Diverse Ethnocultural Backgrounds; PHQ-9 = Patient Health Questionnaire-9; RCT = Randomized Controlled Trial; RFT = Randomized Factorial Trial; SD=Standard Deviation.

1 Welch Two Sample *t*-test; Pearson's Chi-squared test.

In addition, White participants were more likely living in a smaller town or village and reported lower depressive symptom levels at prescreen (T0). Despite the inclusion criterion of mild PHQ-9 or GAD-7 scores, some patients showed none-to-minimal values at prescreen (T0), because they met criteria in only one score (e.g., mild in PHQ-9, minimal in GAD-7). Gender distributions were comparable across samples. As shown in the heatmap in Online Supplement 4, engagement and adherence variables were generally moderately positively correlated, with the strongest associations observed among number of logins, days with platform access, and lesson completion. Correlations remained below levels indicative of multicollinearity (all *r* < 0.80), and therefore did not pose concerns for mediation analyses.

### 3.2 Findings RQ1: group differences in symptom change, engagement, and adherence

#### 3.2.1 RQ1.1 symptom change comparison

**Fig. 2:**
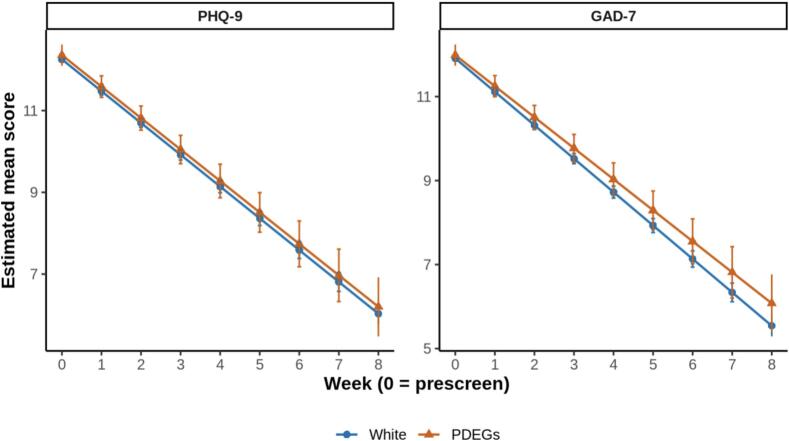
*Notes.* Lines depict adjusted predicted mean PHQ-9 and GAD-7 scores across the 8-week iCBT program (week 0 = prescreen, T0) for White participants and PDEGs, estimated from linear mixed-effects models with random intercepts for participants. Error bars represent 95% confidence intervals around the fixed-effect predictions. Models included fixed effects for week, ethnicity, their interaction, age, dataset, and prescreen symptom severity (PHQ-9 and GAD-7 at prescreen, T0), with prescreen severity and age held at their sample means for prediction. Estimates are based on all available weekly observations and are therefore not restricted to complete cases. GAD-7 = Generalized Anxiety Disorder-7; PDEGs = People of Diverse Ethnocultural Backgrounds; PHQ-9 = Patient Health Questionnaire-9; White = White iCBT participants.

Across the 8-week iCBT program, depressive and anxiety symptoms decreased over time in both White participants and PDEGs, as illustrated by the adjusted model-based trajectories in Fig. 2. Linear mixed-effects models including fixed effects for week, ethnicity, their interaction, prescreen symptom severity, dataset, and age, with participant-level random intercepts and random slopes for week indicated a strong main effect of week for both outcomes, reflecting decreasing PHQ-9 and GAD-7 scores (both *p*s < 0.001). Full model estimates are reported in Online Supplement 5.

For depression (PHQ-9), there was no statistically significant main effect of ethnicity (*b* = 0.10, SE = 0.14, *p* = .46, [−0.17, 0.38]), nor evidence of differential change over time between PDEGs and White participants, as indicated by a non-significant ethnicity × week interaction (*b* = 0.01, SE = 0.05, *p* = .86, [−0.09, 0.10]). However, prescreen PHQ-9 was strongly associated with symptom levels (*b* = 0.84, SE = 0.01, *p* < .001, [0.83, 0.86]). As depicted in Fig. 2, adjusted predicted trajectories showed parallel declines in PHQ-9 scores for both groups from prescreen (T0) to post-intervention assessment (T2).

A similar pattern was observed for anxiety symptoms (GAD-7). Week was significantly associated with decreasing symptoms (*b* = −0.80, SE = 0.02, *p* < .001, [−0.83, −0.77]), and prescreen GAD-7 severity was strongly related to symptom levels (*b* = 0.82, SE = 0.01, *p* < .001, [0.80, 0.84]). Neither the main effect of ethnicity (*b* = 0.08, SE = 0.14, *p* = .58, [−0.19, 0.35]) nor the ethnicity × week interaction (*b* = 0.06, SE = 0.05, *p* = .22, [−0.03, 0.15]) reached statistical significance, indicating similar rates of change in GAD-7 scores across groups. Detailed analyses of post-intervention missingness and clinically significant improvement are reported in Online Supplement 6 and 7.

#### 3.2.2 RQ1.2 engagement and adherence comparison

Because engagement outcomes were right-skewed, results are presented as adjusted mean differences with HC3 robust CI rather than standardized effect sizes. Adjusted comparisons of engagement metrics showed small but consistent group differences (see Online Supplement 8). PDEGs sent fewer messages than White participants (adjusted mean difference = −0.7, 95% CI [−1.0, −0.4]) but had slightly more phone conversations (adjusted mean difference = 0.2, 95% CI [0.02, 0.4]). Standardized effect sizes are not reported because engagement outcomes were skewed count variables and analyses focused on adjusted mean differences with robust CI. Other engagement indicators, including days of platform access, number of logins, and messages received, did not differ significantly.

Program completion also differed modestly between groups (see Online Supplement 9). After adjustment for age, dataset, and prescreen (T0) PHQ-9 and GAD-7, PDEGs were less likely to complete the program (≥4 lessons) than White participants (69.3% vs. 63.2%; adjusted difference = −6.1 percentage points, 95% CI [−11.6, −0.7]), corresponding to 25% lower odds of program completion (OR = 0.75, 95% CI [0.58, 0.96]) and a lower adjusted completion probability (RR = 0.91, 95% CI [0.84, 0.99]). Accordingly, PDEGs completed slightly fewer lessons (adjusted difference = −0.35 lessons, 95% CI [−0.56, −0.14]) compared to White participants.

**Fig. 3:**
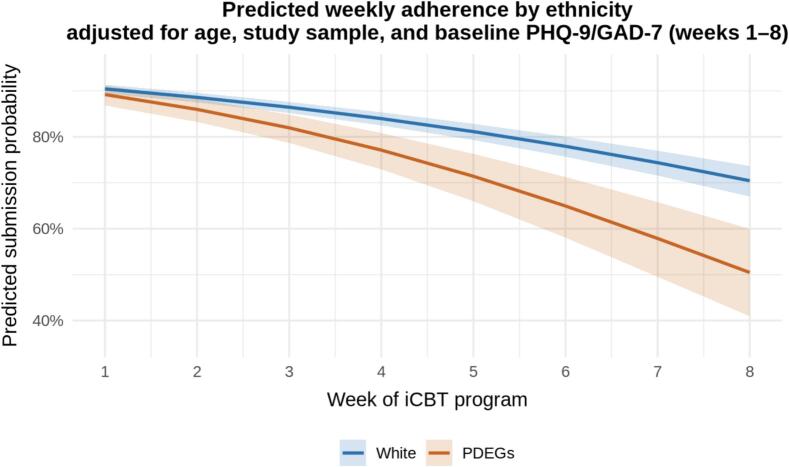
***Notes.*** Predicted weekly adherence to symptom monitoring by ethnicity from a logistic mixed-effects model. Lines show the predicted probability of submitting at least one PHQ-9 or GAD-7 score in each program week for White and PDEGs participants, holding age, dataset, and prescreen (T0) PHQ-9 and GAD-7 scores at their sample means. Shaded bands represent 95% CI based on fixed-effects predictions. The model included week (linear), ethnicity (PDEGs vs White participants), their interaction, age, and prescreen (T0) PHQ-9 and GAD-7 as fixed effects, with random intercepts and random slopes for week at the participant level. CI=Confidence Intervals; GAD-7 = Generalized Anxiety Disorder-7; PDEGs = People of Diverse Ethnocultural Backgrounds; PHQ-9 = Patient Health Questionnaire-9; White = White iCBT participants.

Weekly adherence measured by weekly questionnaire submission rates decreased over the course of the 8-week program for both ethnocultural groups. However, PDEGs showed consistently lower adherence than White participants. As illustrated in Fig. 3, predicted submission probabilities at week 1 were high for both groups (>85%), but the trajectories diverged over time. Model-based predictions indicated an increasing gap across weeks, with a steeper decline among PDEGs. By week 8, predicted questionnaire submission probability was approximately 70% for White participants and 50% for PDEGs.

Results from the generalized linear mixed-effects model, which adjusted for age, dataset, and prescreen (T0) PHQ-9 and GAD-7 scores, indicated significant main effects of week (OR = 0.64, 95% CI [0.60, 0.68]) and ethnicity (OR = 0.61, 95% CI [0.47, 0.78]), showing that adherence decreased over time and that PDEGs had lower odds of weekly submission than White participants (Online Supplement 10). The significant ethnicity × week interaction (OR = 0.79, 95% CI [0.68, 0.92]) further demonstrated that PDEGs' adherence declined more steeply across program weeks. Higher age was associated with increased adherence (OR = 1.37, 95% CI [1.27, 1.49]), whereas higher prescreen PHQ-9 severity was associated with lower adherence (OR = 0.82, 95% CI [0.75, 0.89]), while prescreen GAD-7 was not clearly associated with weekly adherence (OR = 1.08, 95% CI [1.00, 1.18]). In sum, these findings indicate a widening adherence gap over time, with PDEGs showing lower and more rapidly declining engagement with weekly symptom monitoring than White participants.

### 3.3 Findings RQ2: mediation of ethnocultural differences in treatment outcome through engagement and adherence

Because ethnocultural differences in post-intervention (T2) PHQ-9 and GAD-7 scores were small and often nonsignificant (see RQ1.1), and group differences in engagement and adherence were modest (see RQ1.2), the preconditions for mediation were limited. Accordingly, we conducted all mediation analyses as exploratory

Across complete-case and imputed models, no engagement mediator (days with platform access, number of logins, messages sent, messages received, or phone conversations) showed evidence of a significant indirect effect (ACME) on post-intervention PHQ-9 or GAD-7 (T2; all ACMEs≈0, 95% CIs including 0). Estimates were consistent across complete, unadjusted, and adjusted for age, dataset, and prescreen (T0) symptom severity (Online Supplement 11).

Similarly, none of the adherence mediators (weeks with any PHQ-9 or GAD-7 submission, program completion ≥4 lessons, or total lessons completed) demonstrated significant indirect effects on either outcome (Online Supplement 12). ACME estimates were small and their CIs overlapped zero across all analytic specifications (complete cases, MI unadjusted, MI adjusted). Overall, engagement and adherence indicators did not meaningfully account for ethnocultural differences in post-intervention symptom outcomes within the tested ANCOVA mediation models.

### 3.4 Additional analysis

Exploratory subgroup comparisons relative to White participants are shown in Online Supplement 13, 14, and 15. Of note, we did not include dataset as a covariate in subgroup analyses because most subgroups were represented by a single dataset, precluding meaningful adjustment. Adjusted mean differences in post-intervention PHQ-9 and GAD-7 scores were small across ethnocultural subgroups. First Nations, Inuit, and Métis participants showed modestly higher symptom levels and slightly lower engagement and lesson completion, whereas Asian participants showed modestly lower symptom levels. Other subgroup differences were minimal and confidence intervals generally spanned the null. Overall, differences in symptoms, engagement, and adherence were limited.

## 4 Discussion

This study investigated ethnocultural differences in symptom change, engagement, and adherence in a therapist-guided iCBT program within OTU in Saskatchewan, Canada. In addition, we explored potential mediating effects of engagement and adherence on ethnocultural differences in treatment outcomes. Overall, results indicate that both participants identifying as White and as PDEGs experienced substantial improvements in depressive and anxiety symptoms. At the same time, small but consistent ethnocultural differences emerged in engagement and adherence, particularly weekly questionnaire submission.

Exploratory mediation analyses did not indicate a mediating role of engagement or adherence metrics in explaining ethnocultural differences in post-intervention depressive or anxiety symptoms. In addition, exploratory subgroup analyses suggested only modest differences in symptom change, engagement, and adherence across ethnocultural groups. However, these findings should be interpreted cautiously, as subgroup sample sizes were small and limited statistical power and generalizability.

Importantly, PDEGs comprised only 12% of the analytic sample, which is below their representation in the Saskatchewan population, where approximately 17% self-identify as First Nations, Inuit, and Métis and 14.4% of residents belong to other ethnocultural groups (Sapkota et al., 2023; Saskatchewan Bureau of Statistics, 2022). This is consistent with prior reports of lower participation of PDEGs in iCBT services in the province (Sapkota et al., 2024; Hadjistavropoulos et al., 2022a).

### 4.1 Symptom change and interpretation of ethnocultural similarities

Both White participants and PDEGs demonstrated symptom improvement (from prescreen (T0) to post-intervention (T2) at week 8), although PDEGs entered treatment with slightly higher depressive symptom severity. Unadjusted between-group differences at treatment completion were small and nonsignificant. Our results align with previous findings (Cheng et al., 2019; Owusu et al., 2023) suggesting comparable depressive and anxiety symptom reduction in PDEGs and White participants.

One explanation for the similar outcomes is that PDEGs in this sample may have been relatively well integrated into the Canadian cultural and healthcare context (Berry and Hou, 2017). Given that PDEGs were younger and more likely to be students, the sample may have included more second- or later-generation individuals or younger immigrants with greater exposure to Western mental health models and varying language proficiency (Cheung et al., 2011), potentially reducing barriers to standardized iCBT. However, this interpretation is speculative, as migration history and language proficiency were not assessed, limiting examination of within-group heterogeneity. Future research should measure migration-related and contextual factors to clarify their role in shaping iCBT accessibility and effectiveness across diverse populations.

In exploratory subgroup analyses, First Nations, Inuit, and Métis participants showed somewhat lower engagement and adherence relative to White participants. These differences should not be interpreted as reflecting inherent group characteristics, but rather may point to broader structural, historical, and contextual factors influencing DMHI engagement. Such factors may include systemic discrimination, historical trauma, and differential trust in health-care systems (see Graham et al., 2021; Saskatchewan Bureau of Statistics, 2022).

Taken together, these findings suggest that similar outcomes across broad ethnocultural groups do not preclude important contextual barriers for specific subgroups, and that culturally responsive and structurally informed approaches may be necessary to address persistent inequities in digital mental health care.

### 4.2 Engagement, adherence, and participation over time

Despite similar symptom trajectories, PDEGs exhibited small differences in engagement compared with White participants. PDEGs sent fewer therapist messages but participated slightly more in phone conversations than White participants, suggesting differences in communication patterns or clinician outreach rather than lower engagement. This pattern may reflect cultural differences in help-seeking behaviors or comfort with written versus verbal expression, highlighting the importance of offering flexible modes of therapist contact within digital interventions. It may also reflect OTU practices encouraging therapists to call clients when they have not logged in for a week or completed the weekly questionnaire. Phone calls were unscripted and not matched by clinician–patient ethnocultural background. Because only the number of phone contacts was available, we could not assess whether telephone support differed qualitatively between groups or influenced outcomes.

More pronounced differences were observed in adherence to weekly symptom monitoring. Completion of weekly PHQ-9 and GAD-7 questionnaires was required to access subsequent lessons. Thus, non-completion likely reflected disengagement from program content and may indicate early dropout. However, the extent to which symptom monitoring captures engagement with therapeutic content independently of access to program materials cannot be determined from these data.

Although declining adherence over time was evident in both groups, PDEGs demonstrated lower overall submission probabilities and a steeper decline across treatment weeks compared with White participants. This is in line with previous findings by Solar et al. (2021). These differences in program adherence persisted after adjustment for age, dataset, and prescreen symptoms. Importantly, adherence gaps widened over time, suggesting that initial uptake does not guarantee sustained engagement for PDEGs. At the same time, lower observed program adherence, particularly questionnaire submission, may not necessarily reflect lower therapeutic involvement, but could also indicate differences in available time, competing demands, or resources.

### 4.3 Absence of mediation and implications for mechanisms of change

We found no evidence that engagement or adherence mediated ethnocultural differences in post-intervention (T2) PHQ-9 or GAD-7 outcomes in our exploratory mediation analyses. Indirect effects were consistently small and nonsignificant across complete-case and multiple-imputation analyses. These findings do not provide clear evidence that the quantity of engagement or adherence, explains ethnocultural differences in treatment outcomes.

One interpretation is that iCBT may be resilient to moderate variability in engagement and adherence, allowing participants to benefit even when usage is lower or declines over time. This interpretation is supported by Donkin et al. (2013), who found that general usage metrics were not consistently associated with symptom improvement in DMHI. However, higher module completion was linked to positive treatment outcomes, consistent with Gan et al. (2021). Alternatively, qualitative aspects of engagement, such as perceived content relevance or therapeutic alliance, may play a more important role than frequency-based indicators. Lastly, the lack of mediation should also be interpreted in light of the small total effects observed, which constrain detection of indirect pathways.

### 4.4 Selection effects and unobserved barriers to access

Notably, this study only included PDEGs who initiated and engaged with iCBT. The higher baseline depressive symptom severity observed among PDEGs may also reflect differences in help-seeking, referral pathways, or timing of treatment initiation. Individuals facing barriers, such as limited English proficiency, lower digital literacy, unreliable internet access, greater mistrust in digital or mental health services, or limited access to healthcare providers and referral pathways, may have been less likely to learn about the OTU, receive a referral, or enroll in the program (Chiu et al., 2018; Williams et al., 2024; Lorenzo-Luaces et al., 2024; Stiles-Shields et al., 2022; De Jesús-Romero et al., 2024). As a result, these groups may be underrepresented in the analytic sample. Consequently, PDEGs included in this study may represent a relatively advantaged subset, which could partially explain similarity in engagement and treatment outcomes between groups.

### 4.5 Strengths and limitations

This study has several strengths. First, it examined a large, naturalistic clinical sample using a validated, therapist-guided iCBT program, enhancing ecological validity. Second, ethnicity data were available for most participants, enabling systematic comparison between PDEGs and White participants. Third, engagement and adherence were conceptualized as multifaceted constructs, incorporating indicators such as number of logins, therapist communication, symptom monitoring, weekly submissions, lesson completion, and program completion, providing a nuanced picture of iCBT participation. Fourth, the use of linear models and GLMM enabled the examination of symptom trajectories and program adherence over time while accounting for within-participant correlation and differential observation patterns, thereby maximizing use of repeated-measures data. Finally, to our knowledge this is one of the first studies to systematically compare symptom change, engagement, and adherence to an iCBT program between PDEGs and White participants.

However, this study faces several limitations. First, the PDEG group was heterogeneous, comprising patients from different ethnocultural backgrounds, with small subgroup sizes limiting generalizability to specific ethnocultural groups. Aggregation was necessary for statistical power but may have masked meaningful subgroup differences. Future studies should prioritize sufficiently powered analyses of individual ethnocultural groups. Second, engagement was assessed using aggregate counts per patient, which precludes conclusions about the timing or distribution of engagement across the program. Consequently, participants may have completed the intervention over shorter or longer periods, with differing lesson-completion patterns. Moreover, engagement and adherence indicators may have overlapped temporally with post-intervention (T2) assessment, such that temporal ordering between the proposed mediators and outcomes could not be fully determined. Third, engagement and adherence measures were limited to quantitative indicators that may not capture qualitative aspects of treatment engagement/experience (e.g., phone conversations).

Fourth, post-intervention data were more frequently missing among PDEGs than White participants. Although mixed-effects models use all available data under a missing-at-random assumption, outcome availability may influence week-8 analyses. However, missingness appeared more closely related to observed engagement and adherence than to ethnocultural group membership, supporting a missing-at-random mechanism conditional on measured variables. Fifth, the observational design precludes causal inference of engagement, adherence, and treatment outcomes. Sixth, outcomes were limited to PHQ-9 and GAD-7 measures. We did not test measurement invariance across time or ethnocultural groups for either measure. Recent work has questioned the temporal stability of the PHQ-9, which may affect interpretation of symptom trajectories and mediation analyses (Hlynsson et al., 2025), although both are well-validated instruments (Kroenke et al., 2001; Spitzer et al., 2006). Finally, the absence of data on non-participants limits conclusions regarding broader access and equity in iCBT delivery.

## 5 Conclusions

In summary, PDEGs who accessed and initiated therapist-guided iCBT appear to benefit comparably to White participants, consistent with outcomes in this selected clinical sample (Sapkota et al., 2024). However, because PDEGs were analyzed as a combined group due to small subgroup sizes, this finding should be interpreted cautiously, as aggregation may have obscured subgroup-specific differences. Moreover, PDEG underrepresentation suggests possible selection bias, indicating that findings may primarily apply to those who accessed and initiated iCBT. Although we did not find substantial evidence for ethnocultural differences in overall engagement or symptom change, PDEGs completed fewer lessons. Preliminary exploratory subgroup analyses suggested some variation across ethnocultural groups, but small subgroup sizes limited interpretation. Future research should incorporate contextual information (e.g., language proficiency, migration history, and structural constraints) and qualitative or process-oriented measures to better understand access and sustained engagement within specific ethnocultural groups. Efforts to culturally adapt iCBT may therefore need to address not only content but also program structure and length. Finally, addressing barriers to initiation and ongoing engagement will be essential to ensure that DMHIs reduce, rather than widen, mental health disparities.

## CRediT authorship contribution statement

NR contributed to the conceptualization of the study, conducted the literature review, designed the secondary analysis, carried out and interpreted the data analyses, drafted the manuscript, and approved the final version for publication. HDH was involved in data collection, contributed to the literature review, critically reviewed the manuscript, and provided substantial input during revisions. MN participated in data collection and contributed important feedback through critical revisions of the manuscript. RL co-supervised the project, offered continuous critical guidance, and contributed to shaping the final version of the manuscript. GM contributed to the design of the secondary analysis, supervised the project, provided ongoing critical input, and played a central role in refining the final manuscript. GM serves as the guarantor of the work, assumes full responsibility for its integrity, had full access to the data, and made the final decision regarding publication. Was used to and was accessed via the online ChatGPT platform.

## Declaration of Generative AI and AI-assisted technologies in the writing process

During the preparation of this work the authors used ChatGPT-5.2 accessed via the online ChatGPT platform in order to assist with language editing, stylistic refinement, and adjustment to journal word limits. After using this tool/service, the authors reviewed and edited the content as needed and take full responsibility for the content of the published article.

## Funding

This study was funded by the Hasler Foundation (Grant-No: 23004). The funding source was not involved in the design or conduct of the study, in the collection, management, analysis or interpretation of the data, or the preparation, review, or approval of the manuscript.

## Declaration of competing interest

GM has received funding from the 10.13039/501100004339Stanley Thomas Johnson Stiftung and Gottfried und Julia Bangerter-Rhyner-Stiftung (projects PC 28/17 and PC 05/18), Gesundheitsförderung Schweiz (project 18.191/K50001), the Health Department of the Canton of Basel-Stadt, the 10.13039/501100004362Swiss Heart Foundation (project FF21101), the Research Foundation of the International Psychoanalytic University (IPU) Berlin (projects 5087 and 5217), and the German Federal Ministry of Education and Research (budget item 68606). Additional funding was received in the context of a Horizon Europe project supported by the Swiss 10.13039/501100007352State Secretariat for Education, Research and Innovation (SERI; contract 22.00094). Together with NR, GM received funding from Wings Health in the context of a proof-of-concept study and from the Hasler Foundation (project 23004). GM is a co-founder and shareholder of Therayou AG, which operates in the field of digital and blended mental healthcare. NR is on the board of directors and a shareholder of Therayou AG. GM also receives royalties as an author from publishing companies, including for a book published by Springer, and has received an honorarium from Lundbeck for speaking at a symposium. In addition, GM is compensated for providing psychotherapy, acting as a supervisor, serving as a self-experience facilitator (*Selbsterfahrungsleiter*), and for postgraduate training of psychotherapists, psychosomatic specialists, and supervisors. HDH is employed by the University of Regina and serves as Executive Director of the Online Therapy Unit, which has received funding from the Saskatchewan government, the 10.13039/501100000106Saskatchewan Health Research Foundation, the 10.13039/501100000024Canadian Institutes of Health Research (grants 293379 and 152917), and the Rx&D Health Research Foundation. The funders had no role in the design of this study or in the collection, analysis, or interpretation of the data.

## Data Availability

The dataset analyzed in this study is not publicly available due to privacy protections but can be obtained from the University of Regina upon reasonable request and approval by the REB.
