# Supplementary material for: Ethnocultural differences in symptom change, engagement, and adherence in internet-delivered cognitive behavioral therapy: A secondary analysis of a healthcare-embedded longitudinal study
Source: Internet Interv. 2026 Jul 9;45:100974. doi: 10.1016/j.invent.2026.100974 (PMC13379997; doi:10.1016/j.invent.2026.100974)
Supplement: Supplementary file 1 — Supplementary material [file mmc1.docx]

Online Supplemental Material

**Online Supplement 1**

**Online Therapy Unit (OTU): The Wellbeing Course**

*Overview of the Wellbeing Course*

The Wellbeing Course offered by the OTU is a transdiagnostic, guided iCBT intervention designed to treat symptoms of anxiety and depression [1,2]. By targeting shared features of these conditions, it addresses their high comorbidity more efficiently than disorder-specific approaches. The program consists of five sequential lessons spanning over 8 weeks that provide psychoeducation on anxiety, depression, and the symptom cycle (Lesson 1), introduces cognitive strategies for identifying and modifying unhelpful thoughts (Lesson 2), teaches skills for managing physiological symptoms (Lesson 3) and problematic behaviors (Lesson 4), and concludes with a summary of core skills alongside guidance for treatment completion and relapse prevention (Lesson 5).

*Program Structure and Lesson Access*

See Figure 1 in the manuscript for details on program procedures. Although participants were required to complete the lessons sequentially, they were allowed to progress at a slower pace if needed. Importantly, lesson access was governed by the time since program start, as well as lesson completion. This means that if participants fell behind, they could catch up, for example, if Lesson 2 was completed on day 23, Lesson 3 became available immediately afterward without an additional waiting period. Weekly symptom questionnaires were required for lesson access and were administered according to the current program week. These questionnaires were time limited and expired if not completed within the scheduled week. Participants were required to complete the questionnaire corresponding to the current program week before accessing the lesson they were working on and were not required to retrospectively complete questionnaires from earlier weeks if these had been missed. Consequently, some participants completed only a subset of the weekly questionnaires during the intervention period.

*Screening and Assessment Procedures*

The OTU program initiates with an online screening process, during which participants provide information on sociodemographic characteristics, mental health status, and healthcare utilization through standardized questionnaires, such as the Patient Health Questionnaire-9 and the Generalized Anxiety Disorder-7 [3,4]. Eligible individuals are then invited to a telephone interview to confirm their suitability for the iCBT intervention, while ineligible interviewees are directed to alternative support services. Throughout the 8-week intervention, participants had to complete weekly self-reported symptom measures, namely the PHQ-9 and GAD-7, in order to be able to continue to work on lesson content. Additional assessments are conducted post-treatment at week 8 with follow-ups after 16 and 26 weeks for participants enrolled in under ClinicalTrials.gov NCT04228575, and at 20 weeks for all other participants. However, this secondary analysis did not include any follow-up data as we focused exclusively on the active intervention period, including online screening (T0) and the post-intervention assessment (T2) at 8 weeks. All questionnaires were administered online on the OTU website.

*Therapist Support*

Participants received support from trained clinicians, typically psychologists or social workers, who had completed OTU-specific training. Clinicians monitored progress, provided feedback, and offered motivational support. The OTU did not match clinicians to patients based on PDEG background. Therapists generally contacted participants once weekly via secure messaging, spending approximately 15-20 minutes reviewing progress, symptom measures, and participant messages before providing feedback and motivational support. In addition, clinicians could contact participants by telephone when clinically indicated, for example when symptoms increased substantially, suicide risk was suspected, participants had not logged in for a week, had not completed weekly questionnaires, or when a phone call was considered more appropriate for addressing participant concerns. Therapists followed a treatment manual, and regular audit and feedback procedures were used to support adherence to program protocols [5]. Phone calls did not follow a fixed script. Rather, clinicians tailored their content and duration to the reason for contact and participants’ needs. In the present dataset, phone contacts were recorded only as aggregate counts and lacked information on clinician characteristics, timing, duration, or content. Further details about the intervention and therapist support are provided in earlier publications [6].

*References*

1. Titov N, Dear BF, Staples LG, Terides MD, Karin E, Sheehan J, Johnston L, Gandy M, Fogliati VJ, Wootton BM, McEvoy PM. Disorder-specific versus transdiagnostic and clinician-guided versus self-guided treatment for major depressive disorder and comorbid anxiety disorders: a randomized controlled trial. Journal of Anxiety Disorders 2015 Oct;35:88–102. doi: 10.1016/j.janxdis.2015.08.002

2. Hadjistavropoulos HD, Peynenburg V. Fifteen years of internet-delivered cognitive behaviour therapy in Saskatchewan: expanding access to evidence-based care. hcq 2025 Oct 31;28(3):22–29. doi: 10.12927/hcq.2025.27735

3. Kroenke K. Enhancing the clinical utility of depression screening. CMAJ 2012 Feb 21;184(3):281–282. doi: 10.1503/cmaj.112004

4. Spitzer RL, Kroenke K, Williams JBW, Löwe B. A brief measure for assessing generalized anxiety disorder: the GAD-7. Arch Intern Med 2006 May 22;166(10):1092–1097. doi: 10.1001/archinte.166.10.1092

5. Hadjistavropoulos HD, Peynenburg V, Thiessen DL, Nugent M, Karin E, Staples L, Dear BF, Titov N. Utilization, patient characteristics, and longitudinal improvements among patients from a provincially funded transdiagnostic internet-delivered cognitive behavioural therapy program: observational study of trends over 6 years. Can J Psychiatry 2022 Mar;67(3):192–206. doi: 10.1177/07067437211006873

6. Titov N, Dear BF, Staples LG, Bennett-Levy J, Klein B, Rapee RM, Shann C, Richards D, Andersson G, Ritterband L, Purtell C, Bezuidenhout G, Johnston L, Nielssen OB. MindSpot Clinic: an accessible, efficient, and effective online treatment service for anxiety and depression. PS 2015 Oct;66(10):1043–1050. doi: 10.1176/appi.ps.201400477

**Online Supplement 2**

**Detailed Description of Data Management and Statistical Software**

We conducted all data management and modelling procedures using packages from the tidyverse (for data wrangling and visualization), janitor (data cleaning), gt and gtsummary (table generation), forcats (factor handling), lme4 and lmerTest (mixed-effects modelling), broom and broom.mixed (organizing model output), survival (support functions), haven (importing labelled SPSS/Stata/SAS data), stringr (string manipulation), emmeans (estimated marginal means), scales (formatting), and lmtest and sandwich (robust inference).

**Online Supplement 3**

**Exploratory Ethnocultural Subgroup Analyses**

To explore potential heterogeneity within the aggregated PDEG category, we conducted exploratory subgroup analyses comparing individual ethnocultural subgroups with White participants. Given limited subgroup sample sizes, analyses were restricted to groups with ≥20 participants and interpreted cautiously. Separate linear regression models (ANCOVA framework for symptoms) were estimated using complete-case data, with robust HC3 standard errors. Results are presented as mean differences and visualized using forest plots for symptoms, engagement, and adherence.

Symptom models examined differences in week-8 (T2) PHQ-9 and GAD-7 scores and the corresponding prescreen (T0) symptom score (ANCOVA framework). Engagement and adherence models estimated differences in engagement and adherence indicators PHQ-9 and GAD-7 scores.

All subgroup analyses used a complete-case approach, requiring observed post-intervention (T2) PHQ-9 and GAD-7 scores. We interpreted missing values in engagement and adherence variables as indication for the absence of any engagement or adherence and coded them as zero. We used HC3 heteroskedasticity-consistent standard errors to obtain robust 95% CI. Given limited subgroup sample sizes and the exploratory nature of these comparisons, we did not conduct subgroup-specific mediation analyses. Results are reported as adjusted mean differences (subgroup vs. White participants) and visualized using separate forest plots for symptoms, engagement, and adherence.

**Online Supplement 4**

**
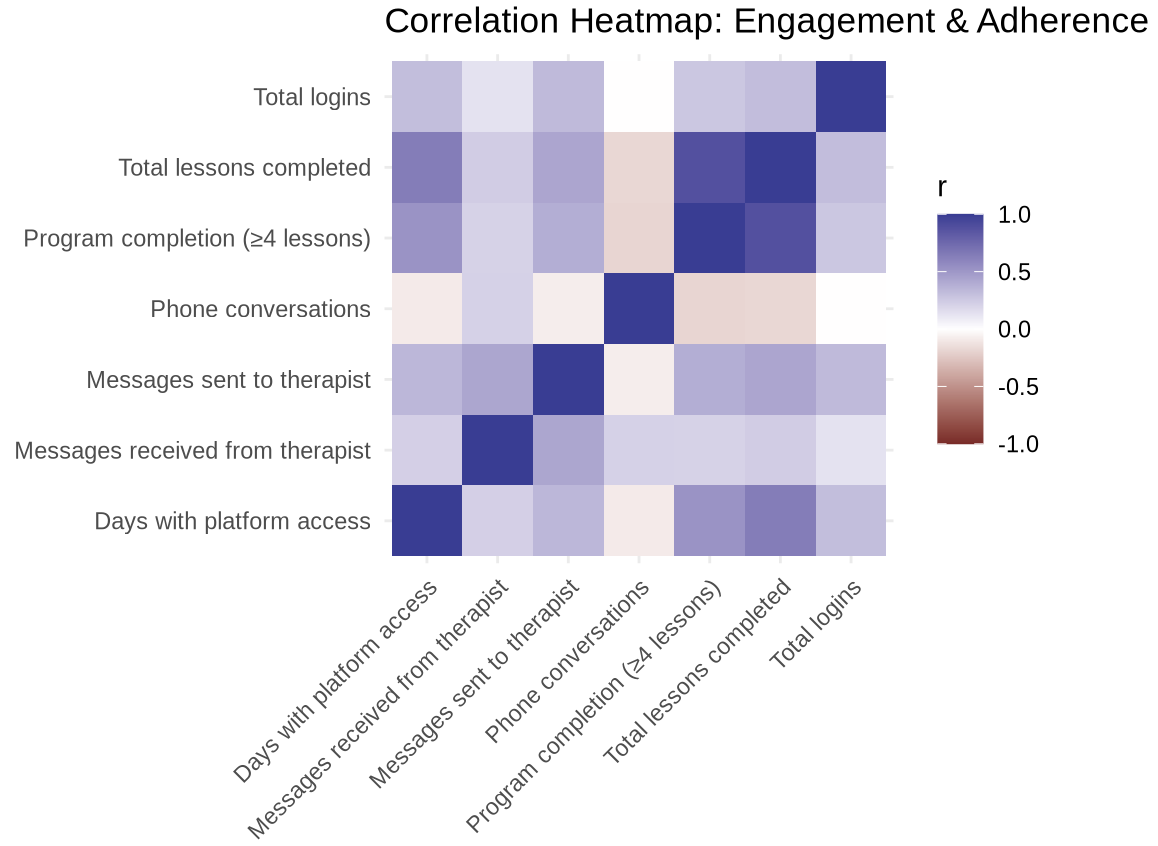
**

***Notes.*** *Correlation heatmap of engagement and adherence variables. Colors represent Pearson correlation coefficients (r), ranging from -1 to +1. Higher correlations indicate behavioral metrics that tend to co-occur (e.g., greater logins and more lessons completed), whereas lighter tones reflect weaker associations. All variables were analyzed in their raw observed form without adjustment for covariates.*

**Online Supplement 5**

| Predictor | *b* | SE | t | df | *p*-values | 95% CI |
| --- | --- | --- | --- | --- | --- | --- |
| **PHQ-9** |  |  |  |  |  |  |
| Intercept | 1.20 | 0.19 | 6.26 | 2638 | <.001 | [0.82, 1.57] |
| Week | -0.78 | 0.02 | -48.15 | 2085.5 | <.001 | [-0.81, -0.75] |
| Ethnicity (PDEGs vs White participants) | 0.10 | 0.14 | 0.73 | 2754.7 | 0.46 | [-0.17, 0.38] |
| Age | -0.005 | 0.003 | -1.32 | 2607.4 | 0.19 | [-0.01, 0.002] |
| Prescreen PHQ-9 (week 0) | 0.84 | 0.01 | 103.75 | 2662.0 | <.001 | [0.83, 0.86] |
| Dataset (Additional alcohol-related materials OS vs Booster/extension RFT) | -0.02 | 0.10 | -0.19 | 2669.6 | 0.85 | [-0.22, 0.18] |
| Dataset (Sleep RCT vs Booster/extension RFT) | 0.032 | 0.142 | 0.23 | 2668.7 | 0.82 | [-0.25, 0.31] |
| Ethnicity × Week | 0.01 | 0.05 | 0.17 | 2203.6 | 0.86 | [-0.09, 0.10] |
| **GAD-7** |  |  |  |  |  |  |
| Intercept | 1.70 | 0.20 | 8.74 | 2654 | <.001 | [1.32, 2.09] |
| Week | -0.80 | 0.02 | -50.8 | 2119.3 | <.001 | [-0.83, -0.77] |
| Ethnicity (PDEGs vs White participants) | 0.08 | 0.14 | 0.56 | 2751.6 | 0.58 | [-0.19, 0.35] |
| Age | -0.001 | 0.003 | -0.46 | 2607.6 | 0.65 | [-0.01, 0.01] |
| Prescreen GAD-7 (week 0) | 0.82 | 0.01 | 92.88 | 2659.9 | <.001 | [0.80, 0.84] |
| Dataset (Additional alcohol-related materials OS vs Booster/extension RFT) | -0.09 | 0.10 | -0.95 | 2669.8 | 0.34 | [-0.29, 0.10] |
| Dataset (Sleep RCT vs Booster/extension RFT) | 0.04 | 0.14 | 0.27 | 2669.5 | 0.79 | [-0.23, 0.31] |
| Ethnicity × Week | 0.06 | 0.05 | 1.23 | 2242.1 | 0.22 | [-0.03, 0.15] |

***Notes.*** *Results are from linear mixed-effects models predicting depressive (PHQ-9) and anxiety (GAD-7) symptom scores across the 8-week iCBT program (week 0=prescreen). Models controlled for baseline severity of the same outcome (PHQ-9 or GAD-7, respectively). Baseline scores of the non-focal symptom were not included to avoid overadjustment and preserve interpretation of symptom-specific change over time. Models included random intercepts and random slopes for week at the participant level. Fixed effects included week (0-8), ethnicity (PDEGs vs. White participants), the Week × Ethnicity interaction, age, and baseline symptom severity at prescreen (week 0). Degrees of freedom were estimated using the Satterthwaite approximation. Regression coefficients (b) are unstandardized. CI=confidence interval; GAD-7=Generalized Anxiety Disorder-7; OS=Observational Study; PDEGs=People of Diverse Ethnocultural Groups; PHQ-9=Patient Health Questionnaire-9; RCT=Randomized Controlled Trial; RFT=Randomized Factorial Trial; SE=standard error.*

**Online Supplement 6**

**Analyses of Post-Intervention Missingness and Predictors**

Method: To evaluate potential bias due to differential attrition, we compared week 8 (T2) PHQ-9 and GAD-7 missingness between ethnocultural groups using χ² tests and examined predictors of missingness with adjusted logistic regression models, assessing the plausibility of a missing-at-random assumption. We additionally conducted supportive analyses of clinically significant improvement from T0 to T2 to evaluate the robustness of outcome findings.

Results: Missingness of post-intervention outcomes (week 8) differed by ethnocultural group, with PDEGs more likely than White participants to have missing PHQ-9 data (39.3% vs. 28.5%), χ²(1)=15.32, p<.001, and missing GAD-7 data (40.2% vs. 28.8%), χ²(1)=16.89, p<.001. These differences indicated differential outcome availability at post-intervention and were addressed in subsequent analyses using mixed-effects models and multiple imputation. In adjusted logistic regression models, missing PHQ-9 outcomes at week 8 were primarily associated with lower engagement and adherence, including fewer days with platform access (OR=0.97, 95% CI [0.96, 0.98]), fewer phone conversations (OR=0.83, 95% CI [0.74, 0.94]), fewer completed lessons (OR=0.80, 95% CI [0.69, 0.92]), and fewer weeks with any PHQ-9 or GAD-7 submission (OR=0.36, 95% CI [0.32, 0.42]). Prescreen depressive symptom severity showed a small association with missingness (OR=1.03, 95% CI [1.00, 1.07]), whereas ethnocultural group, age, prescreen anxiety symptoms, and dataset were not independently associated with missing PHQ-9 outcomes. Parallel analyses for missing GAD-7 outcomes yielded a similar pattern: dataset was not a significant predictor (Additional alcohol-related materials (OS) vs. Booster/extension RFT (NCT04228575): OR=1.33, 95% CI [0.91, 1.93], Sleep RCT (NCT04512768) vs. Booster/extension RFT (NCT04228575): OR=1.23, 95% CI [0.77, 1.96]), ethnocultural group was not independently associated with missingness (OR=1.14, 95% CI [0.73, 1.77]), and lower engagement and adherence indicators were the strongest predictors of missing GAD-7 data.

**Online Supplement 7**

**Supplementary Analyses of Clinically Significant Change**

Method: To complement trajectory-based analyses and enhance clinical interpretability, we examined rates of clinically significant improvement from prescreen (T0) to post-intervention (T2) across ethnocultural groups using adjusted logistic regression models and within-group change estimates, thereby assessing the robustness and practical significance of treatment effects.

Results: Supportive analyses based on clinically significant improvement from prescreen (T0) to post-intervention (T2) showed comparable proportions of improvement across groups (PHQ-9: 60.5% in White participants vs. 59.2% in PDEGs; GAD-7: 68.0% vs. 66.3%). Logistic regression models adjusted for age, dataset, and prescreen symptom severity indicated no statistically significant differences in the odds of clinically significant improvement between PDEGs and White participants for either outcome (PHQ-9: OR=0.85, 95% CI [0.61, 1.20], p=.35; GAD-7: OR=0.85, 95% CI [0.60, 1.21], p=.37). Within-group analyses further indicated substantial reductions in depressive and anxiety symptoms from prescreen (T0) to post-intervention (T2) in both groups. Mean PHQ-9 scores decreased by 6.26 points in White participants and by 6.09 points in PDEGs, while mean GAD-7 scores decreased by 6.30 and 5.97 points, respectively. All within-group changes were statistically significant (ps< .001).

**Online Supplement 8**

| Metrics | N | White participants (adjusted mean) | PDEGs (adjusted mean) | Adjusted mean difference  (PDEGs vs White participants, 95% CI) | | *p*-values |
| --- | --- | --- | --- | --- | --- | --- |
| Days with platform access | 2559 | 65.3 | 62.3 | -2.5 | [-7.6, 2.6] | 0.34 |
| Number of logins | 2502 | 22.2 | 22.0 | -0.2 | [-3.1, 2.7] | 0.89 |
| Messages sent | 2558 | 2.7 | 2.0 | -0.7 | [-1.0, -0.4] | <0.001 |
| Messages received | 2558 | 6.8 | 6.7 | -0.1 | [-0.4, 0.2] | 0.42 |
| Phone conversations | 2560 | 1.0 | 1.2 | 0.2 | [0.02, 0.4] | 0.03 |

***Notes.*** *Adjusted Comparison of* *Engagement Metrics. Values reflect adjusted mean differences between PDEGs and White participants from linear regression models controlling for age, dataset, and prescreen PHQ-9 and GAD-7 scores. HC3 heteroskedasticity-consistent (robust) standard errors were used for p-values and 95% confidence intervals, which provide bias correction when residual variance is unequal across observations. Positive adjusted differences indicate higher values in PDEGs; negative values indicate higher engagement among White participants. Of note, engagement metrics reflect total counts aggregated per patient across their period of platform use. Engagement metrics represent total counts aggregated per patient and do not reflect the timing or distribution of engagement across the program. Accordingly, patients may have engaged for fewer or more than 8 weeks (e.g., completing all lessons in a shorter period or engaging longer without completing all lessons). CI=Confidence Intervals; GAD-7=Generalized Anxiety Disorder-7; PDEGs=People of Diverse Ethnocultural Backgrounds; PHQ-9=Patient Health Questionnaire-9; SD=Standard Deviation.*

**Online Supplement 9**

| Metrics | White participants (adj. mean) | PDEGs (adj. mean) | Adjusted difference (PDEGs vs White participants, 95% CI) | | OR (PDEGs vs. White participants, adj., 95% CI) | RR (PDEGs vs. White participants, adj., 95% CI) | *p*-values |
| --- | --- | --- | --- | --- | --- | --- | --- |
| Program completion (≥ 4 lessons, adjusted) | 69.3% | 63.2% | -6.1% | [-11.6, -0.7] | 0.75 [0.58, 0.96] | 0.91 [0.84, 0.99] | 0.03 |
| Total lessons completed (mean, adjusted) | 4.10 | 3.75 | -0.35 lessons | [-0.56, -0.14] |  |  | <0.001 |
| Weeks with any PHQ-9 or GAD-7 submitted (mean, adjusted) | 5.84 | 5.37 | -0.47 weeks | [-0.74, -0.20] |  |  | <0.001 |

***Notes.*** *Adjusted estimates from regression models controlling for age, dataset, and prescreen PHQ-9 and GAD-7. Completion ≥4 lessons: adjusted probabilities and risk difference (percentage points) from a linear probability model (LPM). Adjusted OR from logistic regression; adjusted RR from a Poisson model with log link and robust (HC3) SEs. Total lessons and weeks submitted: adjusted mean differences from OLS with HC3 robust SEs. Differences are PDEGs minus White. The core adherence analytic sample with complete covariates comprised White participants n=2276 and PDEGs n=322, row-wise Ns may be slightly smaller due to missing outcome data. GAD-7, Generalized Anxiety Disorder-7; CI=Confidence Intervals; HC3=Heteroskedasticity-Consistent robust standard errors (type 3); OLS=Ordinary Least Squares; OR=Odds Ratio; PDEGs=People of Diverse Ethnocultural Backgrounds; RR=Risk Ratio.*

**Online Supplement 10**

| Predictor | *b* (log-odds) | OR | SE | 95% CI (OR) | *p*-values |
| --- | --- | --- | --- | --- | --- |
| Intercept | 1.32 | 3.76 | 0.07 | [3.28, 4.31] | <.001 |
| Week (standardized) | -0.45 | 0.64 | 0.03 | [0.60, 0.68] | <.001 |
| Ethnicity (PDEGs vs. White participants) | -0.49 | 0.61 | 0.13 | [0.48, 0.78] | <.001 |
| Age (standardized) | 0.32 | 1.37 | 0.04 | [1.27, 1.49] | <.001 |
| Prescreen PHQ-9 (standardized) | -0.20 | 0.82 | 0.05 | [0.75, 0.89] | <.001 |
| Prescreen GAD-7 (standardized) | 0.08 | 1.08 | 0.04 | [1.0, 1.18] | 0.08 |
| Dataset (Additional alcohol-related materials OS vs Booster/extension RFT) | 0.23 | 1.26 | 0.08 | [1.08, 1.48] | 0.004 |
| Dataset (Sleep RCT vs Booster/extension RFT) | 0.14 | 1.15 | 0.11 | [0.92, 1.43] | 0.23 |
| Week x Ethnicity | -0.23 | 0.79 | 0.08 | [0.68, 0.92] | 0.002 |

***Notes.*** *Mixed-Effects Model. The model includes a random intercept and random slope for week at the participant level. Predictors are standardized (mean-centered and scaled). Odds ratios refer to the likelihood of submitting a PHQ-9 or GAD-7 questionnaire in a given week. Number of patients n(White)=2276 and n(PDEGs)=322 included in the mixed-effects model. ORs and 95% CI are based on a mixed-effects logistic regression model with Wald-based inference. CI=confidence interval; GAD-7=Generalized Anxiety Disorder-7; OR=odds ratio; OS=Observational Study; PDEGs=People of Diverse Ethnocultural Backgrounds; PHQ-9=Patient Health Questionnaire-9; RCT=Randomized Controlled Trial; RFT=Randomized Factorial Trial; SE=standard error.*

**Online Supplement 11**

***Notes.*** *Panels A and B display the indirect effects (ACME) of ethnocultural group (PDEGs vs. White participants) on post-assessment PHQ-9 (Panel A) and GAD-7 (Panel B) scores through each engagement mediator. Points represent ACME estimates and horizontal bars indicate 95% confidence intervals. Three analysis types are shown: complete-case mediation, MI without covariate adjustment, and MI adjusted for age, dataset, and prescreen PHQ-9/GAD-7. Positive values indicate higher indirect effects for PDEGs relative to White participants; negative values indicate lower indirect effects. The dashed vertical line marks an effect of zero. ACME=Average Causal Mediation Effect; CI=Confidence Intervals; MI=Multiple Imputation; PHQ-9=Patient Health Questionnaire-9; GAD-7=Generalized Anxiety Disorder-7; PDEGs=People of Diverse Ethnocultural Backgrounds.*

**Online Supplement 12**

***Notes.*** *Panels A and B show the indirect effects (ACME) of ethnocultural group (PDEGs vs. White participants) on post-assessment PHQ-9 (Panel A) and GAD-7 (Panel B) scores through each adherence mediator. Mediators include total lessons completed, program completion (≥4 lessons), and the number of weeks with any PHQ-9/GAD-7 submission. Points represent ACME estimates and horizontal bars indicate 95% confidence intervals. Three analysis types are displayed: complete-case mediation, multiple imputation (MI) without covariate adjustment, and MI adjusted for age, dataset, and prescreen PHQ-9/GAD-7. Positive values indicate higher indirect effects for PDEGs relative to White participants; negative values indicate lower indirect effects. The dashed vertical line denotes an indirect effect of zero. ACME=Average Causal Mediation Effect; CI=Confidence Intervals; MI=Multiple Imputation; PHQ-9=Patient Health Questionnaire-9; GAD-7=Generalized Anxiety Disorder-7; PDEGs=People of Diverse Ethnocultural Backgrounds.*

**Online Supplement 13**


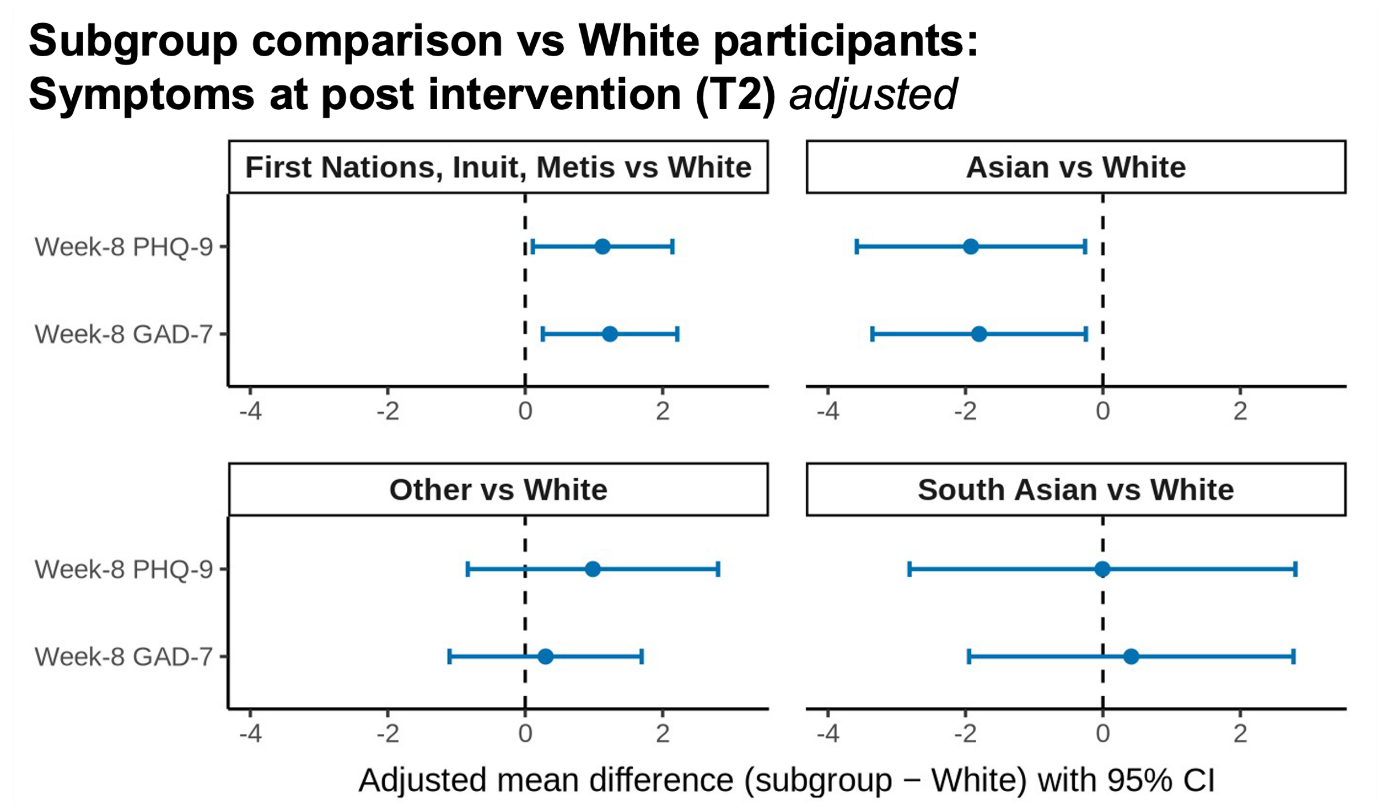


***Notes.*** *Adjusted subgroup comparison vs. White participants: Symptoms at post-intervention (T2). Points show mean differences (subgroup vs. White participants), adjusted for age and prescreen PHQ-9 and GAD-7 scores; bars show 95% CIs. Complete-case models only. CI=Confidence Intervals; GAD-7=Generalized Anxiety Disorder-7; PHQ-9=Patient Health Questionnaire-9.*

**Online Supplement 14**


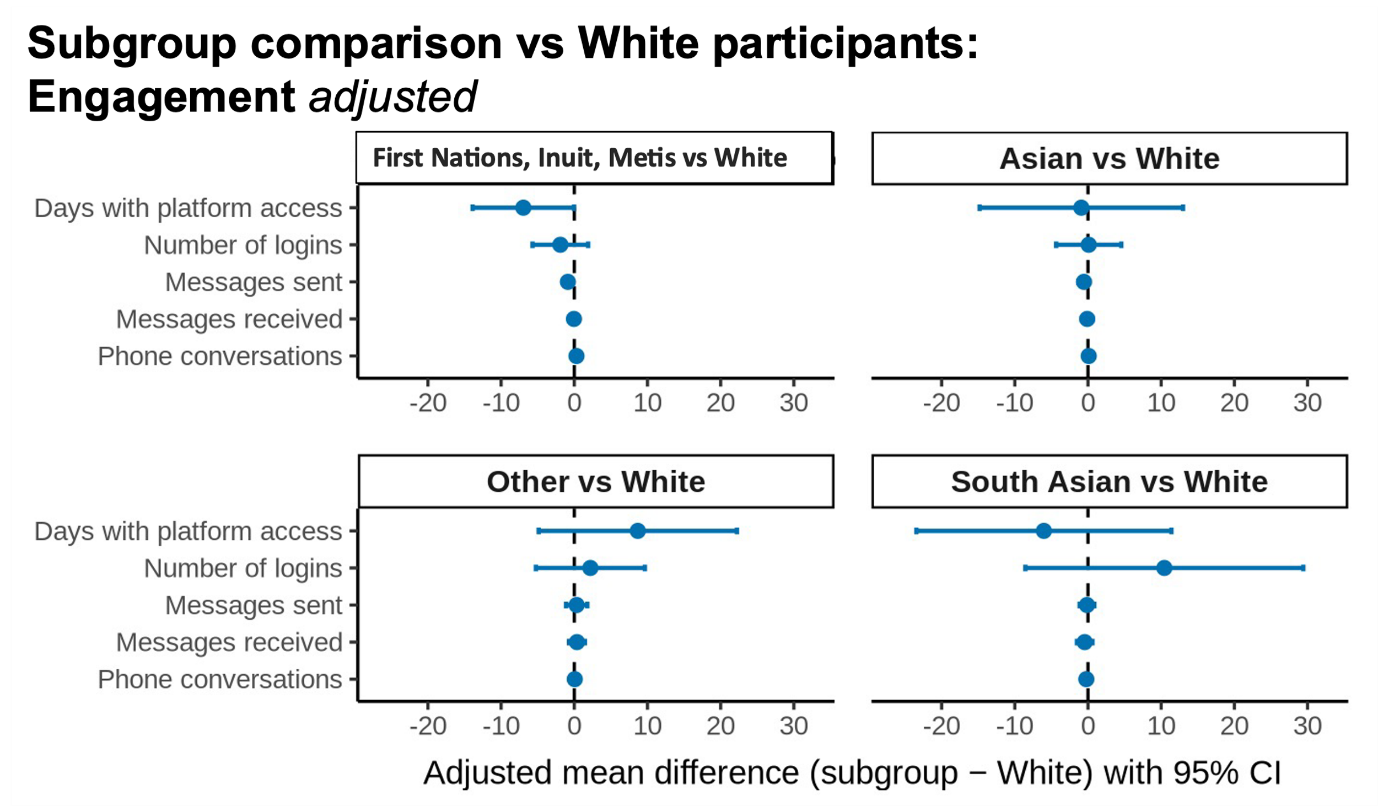


***Notes.*** *Adjusted subgroup comparison vs. White participants: Engagement. Points show mean differences (subgroup vs. White participants), adjusted for age and prescreen PHQ-9 and GAD-7 scores; bars show 95% CIs. Complete-case models only. CI=Confidence Intervals.*

**Online Supplement 15**


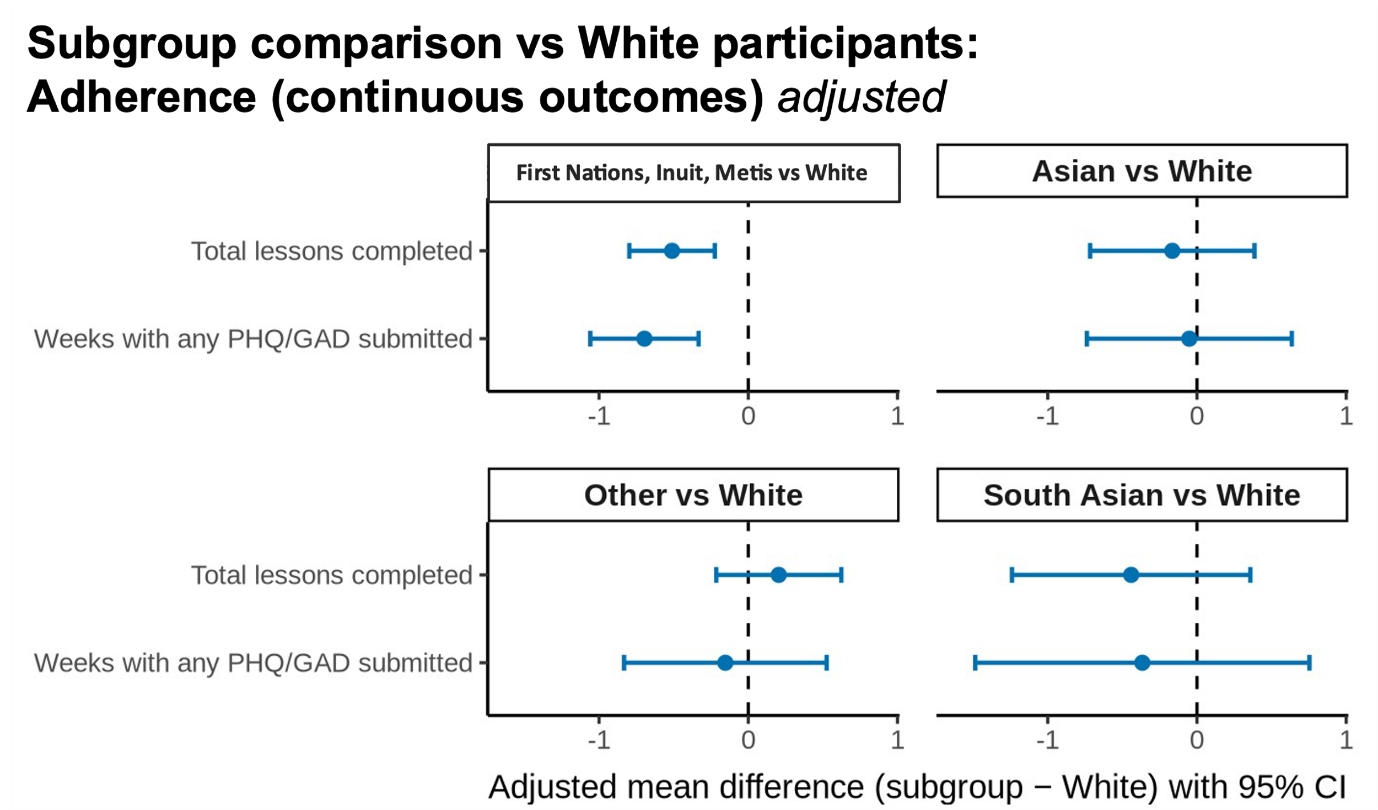


***Notes.*** *Adjusted subgroup comparison vs. White participants: Adherence (continuous outcomes). Points show mean differences (subgroup vs. White participants), adjusted for age and prescreen PHQ-9 and GAD-7 scores; bars show 95% CIs. Complete-case models only. CI=Confidence Intervals; GAD-7=Generalized Anxiety Disorder-7; PHQ-9=Patient Health Questionnaire-9.*
